# Mitochondrial and Nuclear DNA Damage and Repair in Age-Related Macular Degeneration

**DOI:** 10.3390/ijms14022996

**Published:** 2013-01-31

**Authors:** Janusz Blasiak, Sylwester Glowacki, Anu Kauppinen, Kai Kaarniranta

**Affiliations:** 1 Department of Molecular Genetics, Faculty of Biology and Environmental Protection, University of Lodz, Pomorska 141/143, Lodz 90-236, Poland; E-Mail: sglowa@biol.uni.lodz.pl; 2 Department of Ophthalmology, Institute of Clinical Medicine, University of Eastern Finland, Kuopio 70211, Finland; E-Mails: anu.kauppinen@uef.fi (A.K.); Kai.Kaarniranta@kuh.fi (K.K.); 3 Department of Ophthalmology, Kuopio University Hospital, Kuopio 70211, Finland

**Keywords:** age-related macular degeneration, DNA damage, DNA repair, mitochondrial DNA, oxidative stress

## Abstract

Aging and oxidative stress seem to be the most important factors in the pathogenesis of age-related macular degeneration (AMD), a condition affecting many elderly people in the developed world. However, aging is associated with the accumulation of oxidative damage in many biomolecules, including DNA. Furthermore, mitochondria may be especially important in this process because the reactive oxygen species produced in their electron transport chain can damage cellular components. Therefore, the cellular response to DNA damage, expressed mainly through DNA repair, may play an important role in AMD etiology. In several studies the increase in mitochondrial DNA (mtDNA) damage and mutations, and the decrease in the efficacy of DNA repair have been correlated with the occurrence and the stage of AMD. It has also been shown that mitochondrial DNA accumulates more DNA lesions than nuclear DNA in AMD. However, the DNA damage response in mitochondria is executed by nucleus-encoded proteins, and thus mutagenesis in nuclear DNA (nDNA) may affect the ability to respond to mutagenesis in its mitochondrial counterpart. We reported that lymphocytes from AMD patients displayed a higher amount of total endogenous basal and oxidative DNA damage, exhibited a higher sensitivity to hydrogen peroxide and UV radiation, and repaired the lesions induced by these factors less effectively than did cells from control individuals. We postulate that poor efficacy of DNA repair (*i.e.*, is impaired above average for a particular age) when combined with the enhanced sensitivity of retinal pigment epithelium cells to environmental stress factors, contributes to the pathogenesis of AMD. Collectively, these data suggest that the cellular response to both mitochondrial and nuclear DNA damage may play an important role in AMD pathogenesis.

## 1. Introduction

Reactive oxygen species (ROS) and oxidative changes in biomolecules including DNA, have been observed in many diseases, such as cancer, atherosclerosis, diabetes, Alzheimer’s disease, and are believed to be important etiologic factors in these diseases [1]. However, recently it was speculated that ROS and oxidative changes may be the response to the disease, not the cause [2]. Whichever is the case, ROS-related changes to biomolecules are important in the pathogenesis of many serious chronic diseases including ocular disorders such as cataract, glaucoma and age-related macular degeneration (AMD) [3]. In this respect, AMD seems to be of special interest, since aging is a primary factor in its etiology, and oxidative DNA damage may be involved in premature aging [4]. Degenerative changes in retinal pigment epithelium (RPE)—a monolayer of cells between the neural retina and the retinal basement membrane (Bruch’s membrane), resulting from age-related and oxidative stress-induced damage (in RPE cells)—are an anatomical hallmark of AMD [5]. The disease develops in three stages: early, when only a few medium size drusen or other retinal abnormalities are present; intermediate characterized by at least one large druse, several medium sized drusen and geographic atrophy not extending into the center of the macula; and advanced that can be manifested in dry (non-neovascular or nonexudative) or wet (neovascular or exudative) form. In dry form, geographic atrophy extends to the center of macula and choroidal neovascuralization occurs, in wet form, which can lead to subsequent subretinal fluid accumulation, hemorrhage, retinal detachment and fibrotic scar take place [6]. The factors influencing such AMD expansion and the reasons why some patients develop the wet form without preceding dry one are not fully understood. It has been hypothesized that the DNA damage accumulating during aging contributes to vascular dysfunctions [7], which is reflected in changes in the architecture of the retina and choroids, another anatomical hallmark of AMD [8]. This supports the proposal of a link between the age-related variability in DNA repair genes and vascular stiffness [7]. This hypothesis was generalized to state that there was an association between genomic instability and the development of vascular diseases, especially cardiovascular disease, but in this context, genome stability may be seen as equally important in the pathogenesis of AMD.

The human genomic DNA is constantly being damaged by endogenous and exogenous factors, and this attack has consequences on cellular metabolism and defense mechanisms. The function and activity of mitochondria represent a rich source of DNA damage-inducing ROS, which are by-products of the mitochondrial electron transport chain [9]. DNA damage, both to nuclear DNA (nDNA) and its mitochondrial counterpart (mtDNA) normally induces a DNA damage response (DDR) which is crucial for the maintenance of genomic stability. The DDR involves primarily DNA repair which can be combined with or replaced by cell cycle control mechanisms, tolerance to DNA damage including replication bypass, and eventually to triggering cellular death mechanisms: apoptosis, autophagy, and/or mitotic catastrophe [10]. There are different types of DNA repair e.g., the direct reversal of DNA damage, although this is of marginal significance in humans, base excision repair (BER), nucleotide excision repair (NER), the mismatch repair (MMR), non-homologous end joining (NHEJ), homologous recombination repair (HRR), and single strand annealing (SSA). These pathways may operate on their own or in combination, and there is a pronounced difference in their efficacy between the nucleus and mitochondria. If DDR in the retina is expressed as apoptosis, this can led to the death of a population of cells resulting in degenerative retinal changes. Apoptosis can be carried out by the mitochondrial pathway [11]. Since there is clear evidence that mitochondrial damage and dysfunction are associated with the aging retina, and that it is further exacerbated in AMD, studying the DNA damage and repair in mitochondria as the factors contributing to AMD pathogenesis is justified [12,13]. The association between mitochondrial mutagenesis and AMD, as an age-related disease, is in accordance with the Harman mitochondrial theory of aging [14].

## 2. Mitochondrial Mutagenesis

**Figure 1:**
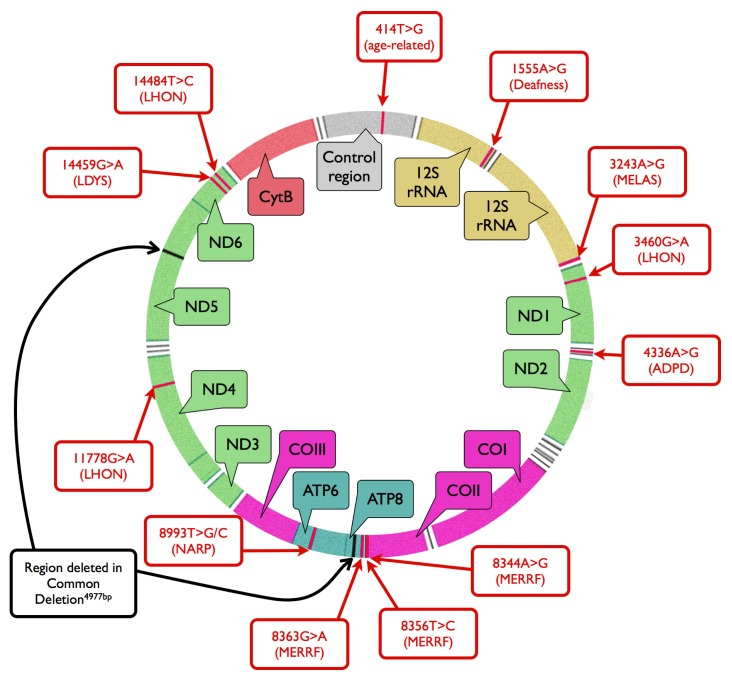
The mitochondrial genome of various organisms, and some mutations in mitochondrial DNA (mtDNA), which can be associated with diseases and aging. The localization of mitochondrial genes is also displayed. White areas denote genes of tRNAs. MELAS–mitochondrial encephalomyopathy; lactic acidosis, and stroke-like episodes; LHON–Leber’s hereditary optic neuropathy; ADPD–Alzheimer’s and Parkinson’s diseases; MERRF–myoclonic epilepsy with ragged red fibers; NARP–neurogenic myopathia, ataxia, retinis pigmentosa; LDYS–LHON + dystonia; rRNA–ribosomal RNA; ND1-6–NADH dehydrogenase subunits; COI-III–cytochrome oxidase subunits; ATP6, −8–ATP synthase subunits; CytB–cytochrome *b*.

The human mitochondrial genome exists in the form of closed double-stranded DNA molecule of 16,569 base pairs [15] (Figure 1). The genome may be targeted by exogenous DNA-damaging agents, including light exposure and ionizing radiations or chemical agents, as well as by endogenous factors, which are products of cellular metabolism. These endogenous factors are mainly ROS, which are formed in the strongly oxidizing environment inside the mitochondria, but they can also be produced by exogenous harmful factors [16]. ROS activity can lead to the production of oxidized derivatives of the DNA bases, including 8-oxoguanine (8-oxoG) [17]. mtDNA may be targeted by the same DNA-damaging factors as nDNA. Thus one can observe the most common forms of DNA damage, *i.e.*, pyrimidine dimers and other products resulting from the UV radiation, ethene adducts formed during the interaction with derivatives of lipid peroxidation, as well as single- and double-strand DNA breaks after exposure to a broad spectrum of factors, both in mtDNA and nDNA [18]. However, it should be noted that UV-induced DNA damage probably may not play a significant role in AMD development as it is usually accepted that UV radiation do not reach the retina and is fully absorbed by cornea and the lens. However, there are some results (revieved in [19]) that suggest possible role of blue light and UV radiation in AMD, especially in cases of chronic exposure. One specific consequence of the action of some of the damaging agents that there is a transient increase in the mtDNA copy number [20]. However, if the amount of damage exceeds levels that can effectively repaired, complete depletion of mtDNA occurs [21].

**Figure 2:**
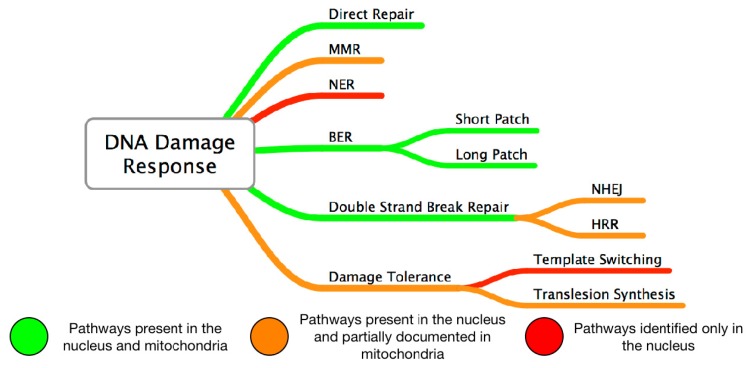
Comparison of DNA damage responses in the nucleus and mitochondria. MMR –mismatch repair; NER–nucleotide excision repair; BER–base excision repair, NHEJ–Non-homologous end joining, HRR–homologous recombination repair.

The extent of DNA damage accumulating in the mitochondrial genome is clearly related to the activity and efficacy of the DNA damage repair pathways [16]. mtDNA was considered to lack DNA repair activity, but multiple repair pathways in mitochondria were revealed, including direct reversal, base excision repair, single-strand break repair, mismatch repair, homologous recombination repair and non-homologous end joining (Figure 2) [22]. All these pathways operate with proteins, which are all encoded by nuclear genes and in principle are similar to those operating in the nucleus, but some differences, are worth noting.

Direct reversal of DNA damage operates in mitochondria in the form of de-alkylation and photoreversal [23,24]. This pathway of mtDNA repair, similarly to its nuclear counterpart is energetically costly and plays a minor role in the maintenance of mitochondrial genome stability. It seems that base excision repair (BER) may be a predominant system of DNA repair dealing with slight DNA lesions induced by hydrolysis, oxidation and alkylation [16]. It may be associated with high exposure of mtDNA to ROS and RNS originating from neighboring electron transport chain [25]. The presence of short patch BER (SP-BER) in mitochondria is well documented and its mechanism is very similar to that operating in the nucleus [26]. Main proteins involved in mitochondrial BER are DNA glycosyales, including UNG1 active on uracil, OGG1 for oxidized guanine products, MYH to remove adenine inserted by DNA polymerase opposite oxidized guanine, NHT1 and NEIL1 for various DNA bases oxidation products [27–29]. The action of mitochondrial glycosylases in SP-BER might be followed by the Ape1 AP endonuclease 1 protein nicking an AP site. DNA polymerase γ and DNA ligase III lead DNA repair synthesis and ligation [30]. The presence of long patch BER (LP-BER) in mitochondria was reported in several studies [31–33]. There are some contradictory results on the involvement of the FEN1 endonuclease, which is critical for LP-BER in the nucleus [22,31]. Several reports point out that FEN1 may be important for mitochondrial LP-BER, closely collaborating with the Dna2 protein, however we do not know yet how is FEN1 transported to mitochondria [34–36].

Mismatch repair (MMR) activity was firstly reported to operate in mitochondria about ten years ago [37]. It does not include MSH2, a key MMR protein in the nucleus and its activity is mainly due to the YBX1, also known as NSEP2 protein [26]. Observed difference in the protein composition of MMR pathways in mitochondria and the nucleus suggests distinct mechanism of this DNA repair system in these two organelles.

Recombinant DNA molecules were identified in several human tissues, providing solid evidence for the presence of recombination in the mitochondrial genome [38]. DNA double strand breaks may be repaired in mitochondria by homologous recombination repair with the forming of D-loop as an intermediate of the repair [39]. Such a loop creates a loose conformation of DNA increasing its accessibility to DNA repair proteins involved in single-strand annealing and recombination. This structure may be also a consequence of replication fork arrest, stimulating recombination events. This all suggests that DNA double strand breaks may be repaired by homologous recombination and non-homologous end joining [40].

Apart from DNA repair, several other actions can be undertaken by the cell in the response to mtDNA damage. They include mtDNA degradation, sanitation of premutagenic triphosphate nucleosides and translesion synthesis, although there is not solid evidence for the latter (reviewed in [41]).

The increased susceptibility of mtDNA to damage compared to that of nDNA does not only result from the differences in the efficacy of DNA repair systems. The lack of a nucleosomal organization in mtDNA facilitates the access of DNA-damaging agents. On the other hand, this property also facilitates the access of DNA repair proteins. mtDNA consists almost exclusively of coding sequences, whereas the contents of such sequences in nDNA is only about 2%. This implies that a damage in mtDNA may potentially evoke more serious consequences in the phenotypic than similar damage in nDNA. Moreover, because mtDNA contains genes encoding proteins of electron transport chain (ETC), damage to these genes may alter the activity of ETC. This altered ETC may contribute to the increased production of ROS, which, in turn, may damage mtDNA. This loop creates so called vicious cycle of events greatly increasing the potential of mtDNA damage [37].

If mtDNA damage is not repaired, it may significantly affect the phenotype. Mutations in mtDNA can cause a number of mitochondrial diseases, and they are also associated with aging and neurodegenerative processes [42,43] (Figure 1). The way in which mutations in mtDNA are phenotypically manifested is much more complex than that of nDNA. Since every mitochondrion contains many copies of mtDNA and each cell usually contains many mitochondria, a single cell may can have different variants of mitochondrial genomes carrying a variety of mutations. This phenomenon is called heteroplasmy [44]. Consequently, there is no controlled segregation of mitochondrial genomes during cell division, analogous to the segregation of nuclear chromosomes, which results in an unequal segregation of mitochondrial genomes containing mutations. This can lead to the existence of significant differences in the frequency and distribution of mutant variants of mitochondrial genes between different cells in the same organism. Certain changes in mtDNA cause symptoms only if the relevant mutations are present in mitochondrial genomes of specific tissues or organs with relevant frequency, often 80% [45,46].

## 3. Mitochondrial DNA Damage and Repair in AMD

Since oxidative stress is one of the major factors in AMD pathogenesis [5], and the main endogenous source of this stress are mitochondria, it is not unreasonable to take a closer look at the role of mtDNA in this disease. Especially when one considers that mtDNA is more susceptible than nDNA to DNA-damaging factors. Although, the mitochondrial genome is very small compared to its nuclear counterpart mitochondria are expressed abundantly in cells with high metabolic rates meaning that there are many copies of mtDNA in every cell [47]. Therefore, mtDNA in retinal cells may be an important target for factors contributing to AMD pathogenesis.

mtDNA may contain deletions which are often associated with various diseases, especially if they accumulate [48]. An accumulation of deletions in mtDNA has been detected in human retina during aging [49]. It should be pointed out that these changes were not observed in the fetus, and thus they may reflect increased mtDNA instability with aging. The stability of mtDNA is attributable mainly to the DDR in mitochondria, which determines the susceptibility of mtDNA to endo- and exogenous factors. These effects have been confirmed in rodent research with the downregulation of DNA repair enzymes involved in repair of oxidative damage occurred in aged RPE and choroid [50]. Therefore, mtDNA damage and repair may contribute to the dysfunction of retina, which in turn, may play a role in AMD. This is the reason for, studying the mtDNA damage and repair.

It was shown that the mtDNA of human RPE cells in culture displayed a greater susceptibility to oxidative DNA damage than the nDNA in these cells [51–54]. Although the results of those experiments indicate that mtDNA of RPE cells is a preferential target of DNA-damaging agents, it was not unequivocally shown that this preference can be attributed to RPE, or in more general to retinal cells. The results of another experiment showing that the mtDNA repair of oxidative damage in RPE cells is slow and inefficient compared to that of nDNA, suggest the general relationship between mutagenesis of mtDNA and nDNA rather than indicate that mtDNA is a preferential target in AMD pathogenesis [55]. The same criticism can be made of the results of an experiment showing an adaptive response to oxidative damage in nDNA of RPE cells, but no corresponding response was observed in mtDNA [52]. However, only RPE cells were investigated no comparison between other cells was made. This issue was clarified in a recent study, which showed that AMD patients had a higher susceptibility of mtDNA damage in the retina rather than in blood [56]. This study also showed that AMD subjects had a high level of large retinal mtDNA deletions and rearrangements in both the coding and non-coding D-loops, and it was speculated that this could have affected the energy production of mitochondria, and the replication and transcription of the mitochondrial genome. However, the number of synonymous and nonsynonymous single nucleotide polymorphisms (SNPs) in the mitochondrial coding genes was similar in AMD and age-matched normal retinas. Since these authors reported previously that the D-loop in AMD retinas had more SNPs than did the normal counterpart [57], it was concluded that disturbances in the regulation of mitochondrial replication and transcription might primarily explain the contribution of mtDNA mutagenesis in the pathogenesis of AMD.

An increased number of lesions in mtDNA of macular region of 46 human donors RPE was observed when they were compared with 26 age-matched subjects [58]. These lesions affected all regions of the mitochondrial genome. There were no changes between AMD donors and controls in the total amount of mtDNA isolated from the eyes.

However, a decrease in total mtDNA has been observed with aging, which is a key pathological factor in AMD. There was no difference in lesion frequency in either mtDNA or nDNA with aging, but in AMD eyes, about an eight times higher frequency of lesions was characterized in mtDNA than in its nuclear counterpart. An increase in lesion frequency in mtDNA was observed with AMD progression evaluated accordingly to Minnesota Grading System (MGS). The authors concluded that the DNA damage in AMD was targeted towards mitochondria and furthermore that the dysfunction of mitochondria induced by a damage to mtDNA, could be linked with AMD.

In a similar study, a positive correlation was observed between the extent of mtDNA damage and the grading level of AMD [59]. Moreover, there was a negative correlation between the efficacy of mtDNA repair after challenging with hydrogen peroxide and the AMD grade. More lesions occurred in RPE cells from the macular region than from the periphery. Furthermore, more mitochondrial heteroplasmic mutations were detected in AMD eyes than in controls. The authors also revealed that the expression of 8-oxoG DNA glycosylase 1 (hOGG1) was reduced in aged RPE cells, especially in macular region, and the expression was further reduced in eyes with increasing MGS values. The authors concluded that the macula-specific impairment in mtDNA repair, increased mtDNA damage, and heteroplasmic mutations were associated with age and AMD severity. These effects may have been associated with a decreased expression level of the *hOGG1* gene or a decreased activity of hOGG1 protein in the macula, or both. The regulation of the expression of the *hOGG1* gene may be tissue or organ-specific, but it is dependent either on nuclear transcription factors or cytoplasmic translation factors and proteins involved in post-translational modifications. The expression can also be organ or site-specific, depending on the accessible factor of its regulation [60]. However, the basic level of expression is determined by the interaction between the gene and the general transcription factors. This interaction is usually sequence-specific, and any damage to the gene may change it, e.g., altering its expression level. Therefore, damage to nDNA may result in an increased risk of mtDNA damage. Several other pathways can be considered to illustrate the general dependence of mtDNA metabolism on the extent of nDNA damage and the efficacy of nDNA damage repair. One of such pathway may be related to efficiency of ATP production in mitochondria affected by nDNA damage, but no such research was performed to date in context of AMD pathogenesis. This reflects the limited autonomy of the mitochondrial genome, and need to be taken into account in considering the role of mtDNA damage in AMD pathogenesis. Therefore, we will take a closer look also at the nuclear DNA, as a possible primary target in molecular pathology of AMD. This view is supported by the lack of NER in mitochondria, the most universal and flexible system of DNA repair. Vascular dysfunction observed in the elderly may be associated with vascular DNA damage [7]. Therefore, disturbances of DNA repair systems may be important in age-related vascular dysfunction. It was reported that some NER genes might be involved in this relationship, but so far there is no direct evidence for the involvement of NER in AMD pathogenesis [7].

## 4. Nuclear DNA Damage and Repair in AMD

One of the most important rationales for considering mutagenesis of mitochondrial DNA as a factor in AMD pathogenesis is relatively low mtDNA repair efficiency and its decline with age, which may have much greater consequences for the cell than the actual changes in mtDNA [61]. Moreover, the regulation of mtDNA metabolism determining its susceptibility to DNA-damaging factors is undertaken by proteins encoded by nDNA, and thus any instability in the nuclear genome will contribute to the damage susceptibility of its mitochondrial counterpart.

ARPE-19, a human retinal pigment epithelial cell line, is a commonly accepted model for research in the molecular mechanisms of AMD pathology [62]. When these cells are exposed to the breakdown products of lutein and β-carotene they displayed nDNA damage contributing to an additional systematic oxidative stress [63]. Oxidative stress, the main factor in AMD pathology, may be associated with many kinds of DNA damage, including single- and double-strand breaks and oxidative modifications of the DNA bases. 8-Oxo-7,8-dihydroguanine (8-oxoG) is one hallmark of oxidative DNA damage and a major promutagenic component in oxidative stress [64]. Oxidation of guanine alters the hydrogen bonding properties of guanine and the glycosidic-preference of guanosine, which may lead to transversion-type mutations [65]. The DNA repair system may combat the detrimental effects of oxidative stress. In most cases, the repair of 8-oxoG is initiated by hOGG1 via the base excision repair (BER) pathway. If 8-oxoG escapes this process, and replicative DNA polymerase misinserts adenine instead of cytosine opposite to 8-oxoG, an alternative pathway of BER will take a place via the hMYH (MUTYH) glycosylase, which is intended to remove the inappropriate adenine. We have recently shown that genetic variability in the *hOGG1* and *hMYH* genes may be associated with the AMD occurrence and progression [66]. It has been reported that the level of 8-oxoG was higher in patients with exudative AMD than in control individuals. This led to the conclusion that DNA damage may be one of the mechanisms underlying the action of oxidative stress in AMD pathology [67].

Several reasons for the difference in the cellular activity to oxidative stress-induced DNA damage between AMD patients and normal individuals need to be considered. For example, do some patients have disturbed expression of DNA repair genes? It was shown that the expression of the *RAD51* gene, the product of which plays a crucial role in the repair of DNA double-strand breaks by homologous recombination, was downregulated in response to an oxidative stimulus in AMD patients [68].

We showed that AMD patients had a higher level of endogenous DNA damage than individuals, who did not report any vision impairment with clinically excluded AMD and that DNA double strand did not contribute to this difference [69]. AMD patients displayed also a higher degree of oxidative DNA damage recognized by DNA base excision repair enzymes NTH1 and Fpg. Lymphocytes from AMD patients exhibited a higher sensitivity to hydrogen peroxide and UV radiation, and repaired the lesions induced by these agents less efficiently than the corresponding cells from the control individuals. We postulate that the impaired efficacy of DNA repair may be combined with the enhanced sensitivity of RPE cells to blue and UV light, contributing to the pathogenesis of AMD [54].

## 5. Discussion

Although the mechanisms behind the pathophysiology of AMD are not fully understood, there is no doubt that oxidative stress plays an important role in this disease. However, there is no consensus about either the source or the direct molecular consequences of stress in RPE cell degeneration and AMD development. Reactive oxygen and nitrogen species produced during stress may damage crucial biomolecules of the RPE cells: proteins, lipids and nucleic acids. It is very difficult to carry out research on the molecular consequences of the stress in RPE cells in human AMD, for example, if one only has access to post-mortem specimens then any results obtained will have very limited value. In general, the direct examination of ocular cells from AMD patients is difficult even impossible for legal reasons, but AMD also appears to have a systemic component and therefore peripheral sites may also manifest the same abnormalities, e.g., genetic changes [68].

**Figure 3:**
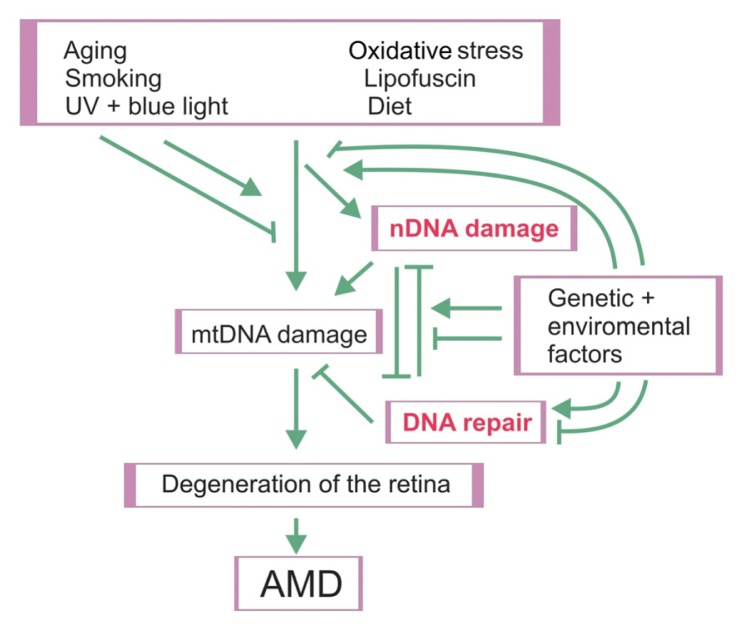
Mutual relationships between AMD risk factors and the cellular reactions that they evoke. mtDNA—mitochondrial DNA, nDNA—nuclear DNA. Arrows indicate stimulation/induction, whereas blunt arrows–inhibition.

There are some experimental results, mentioned above, pointing a role for damage to mtDNA in AMD pathogenesis. The autonomy of the mitochondrial genome, however, is limited and its metabolism is determined mainly by nDNA-encoded proteins. In particular, DNA repair proteins are crucial because the mtDNA repair system is much poorer than its nuclear counterpart. Therefore, damage to nDNA may influence the general metabolism of mtDNA and in particular its susceptibility to DNA damaging factors. This is illustrated in the general scheme of mutual relationships between factors of AMD pathogenesis at the cellular level (Figure 3).

Aging, light exposure, smoking, cardiovascular diseases, unhealthy diet and genes are all factors involved in the increased oxidative stress and accumulation of detrimental lipofuscin in RPE cells [69]. Smoking is statistically significant factor in both types of AMD [70]. More than 60 known carcinogens, including polycyclic hydrocarbons, nitrosoamines, aromatic amines, aldehydes, volatile organic compounds, metals, are contained in tobacco smoke [71]. These compounds may directly or indirectly interact with DNA, inducing various adducts and strand breaks. The smoke contains also unstable free radicals and other ROS and reactive nitrogen species (RNS), which may induce a wide spectrum of oxidative damage to DNA [72]. It was proposed that the mitochondrion was a central target for environmental oxidants, including tobacco smoke [73]. Mitochondrial DNA damage may result in the disturbances of such fundamental cellular processes like energy production, oxidative signaling, immune response and apoptosis [74]. Cigarette smoke exposure was shown to be associated with increased mtDNA damage both *in vitro* and *in vivo* [75–78]. ROS and RNS, which are present in tobacco smoke, were shown to induce a variety of effects, including preferential mtDNA damage in vascular cells [75]. Such mtDNA damage may results in the decrease number of mtDNA replication and in consequence—in decreasing efficacy of assembly of respiratory chain proteins. Second-hand (passive) smoke is often underestimated and not included in studies relating tobacco smoking to diseases. It may also influence mtDNA integrity. It was shown that passive smoke induced mtDNA associated with inactivation of mitochondrial superoxide dismutase, SOD2, in mice [76]. Tobacco smoke includes also benzo-α-pyrene, a strong carcinogen, which may induce adducts with nuclear and mtDNA [79].

The resulting damage of mtDNA may well accelerate RPE degeneration and the development of AMD. Another factor determining these interactions is the variability of DNA repair genes; in this respect, AMD patients may differ from that in the healthy controls [80]. Since aging is associated with enhanced sensitivity to harmful environmental factors and the decreased efficacy of both mitochondrial and nuclear DNA repair capabilities, the well known AMD risk factors may become more harmful as the individual ages. This may be especially important when an individual has a lower than average efficacy of DNA repair. Damage to nuclear DNA may result in the dysfunction of DNA repair proteins and this may contribute to extensive mtDNA damage, which cannot be repaired as efficiently as in the case of when the nuclear genome is intact. Unrepaired damage to nDNA may also induce apoptosis in the retina cells directly contributing to AMD.

## 6. Conclusions

Damage to mitochondrial DNA may play a role in the pathogenesis of AMD, but the susceptibility of mtDNA to DNA-damaging factors is determined mainly by the nuclear DNA. Therefore, both mtDNA and nDNA damage and the efficacy of their repair are obviously important in AMD pathogenesis.
